# Effect of Chemical and Steam Explosion Pulping on the Physical and Mechanical Properties of Sugarcane Straw Pulp Trays

**DOI:** 10.3390/polym15143132

**Published:** 2023-07-23

**Authors:** Kittaporn Ngiwngam, Sinchai Chinvorarat, Pornchai Rachtanapun, Rafael Auras, Thawien Wittaya, Wirongrong Tongdeesoontorn

**Affiliations:** 1 School of Agro-Industry, Mae Fah Luang University, 333 Moo 1 Tasud, Chiang Rai 57100, Thailand; 6251407001@lamduan.mfu.ac.th; 2 Research Group of Innovative Food Packaging and Biomaterials Unit, Mae Fah Luang University, 333 Moo 1 Tasud, Chiang Rai 57100, Thailand; 3 Department of Mechanical & Aerospace Engineering, King Mongkut’s University of Technology North Bangkok, Bangkok 10800, Thailand; sinchai.c@eng.kmutnb.ac.th; 4 Division of Packaging Technology, School of Agro-Industry, Faculty of Agro-Industry, Chiang Mai University, Chiang Mai 50100, Thailand; pornchai.r@cmu.ac.th; 5 Center of Excellence in Agro Bio-Circular-Green Industry (Agro BCG), Chiang Mai University, Chiang Mai 50100, Thailand; 6 Center of Excellence in Materials Science and Technology, Chiang Mai University, Chiang Mai 50200, Thailand; 7 School of Packaging, Michigan State University, 448 Wilson Rd, East Lansing, MI 48824, USA; aurasraf@msu.edu; 8 Faculty of Agro-Industry, Prince of Songkla University, Songkhla 90110, Thailand; thawean.b@psu.ac.th

**Keywords:** sugarcane straw fiber, chemical pulping, steam explosion, hydraulic molding, mechanical properties

## Abstract

Sugarcane straw fiber (SSF) samples were prepared by chemical pulping (CP) and steam explosion (STE). CP (5, 10, 15% NaOH + 0.2% *w*/*w* anthraquinone at 121 °C for 1 h) and STE pressure (1.77, 1.96, and 2.16 MPa at 220 °C for 4 min) SSF trays were molded with a hydraulic hot-press machine at 120 °C, 7 min, and 1.72 MPa. The yield (%) of SSF from STE (54–60% dry basis (db.)) was higher than CP (32–48% db.). STE trays had greater tensile strength than CP. However, STE’s elongation and compression strength was lower than CP tray samples. The trays made from SSF using STE had less swelling in thickness, longer water wetting time, and a higher water contact angle than those made from CP. The micrographs displayed a smaller size of SSF obtained in STE than the CP. The appearance and area of peaks in ATR-FTIR spectra and XRD diffractograms, respectively, revealed that the STE trays had a larger residual lignin content from the lignin study and a lower crystallinity index than the CP trays. Moreover, the lightness values of the STE trays were lower than those of the CP trays due to lignin retention. The study results indicate that CP is the preferred method for producing SSF packaging material with high flexibility and fiber purity. However, when considering the specific SF of 4.28, the STE treatment showed superior physical and mechanical properties compared to CP. This suggests that STE could be an excellent alternative green pulping technique for producing durable biobased trays. Overall, the findings highlight the potential of STE as a viable option for obtaining trays with desirable characteristics, providing a sustainable and efficient approach to tray production.

## 1. Introduction

Sugarcane is abundantly available worldwide, especially in tropical countries, and abundant in lignocellulosic material [1]. Sugarcane production in Thailand fluctuates from year to year. Still, it has generally increased from 94 million tonnes in 2015 to 131 million tonnes in 2019, followed by a decline to 75 million tonnes in 2020, as reported by the FAO in 2022 (Food and Agricultural Organization (FAO), 2022). This rise in sugarcane production corresponds to increased waste generated by the industry. Vasconcelos and Carpio [2] reported that half of the total sugarcane production (consisting of 25% sugarcane bagasse and 25% sugarcane straw) ends up being classified as waste. Sugarcane production in Thailand generates significant biomass, with estimated 34, 33, and 18 million tonnes of sugarcane straws in 2018, 2019, and 2020, respectively (FAO,2022). Burning sugarcane waste in the field causes environmental pollution that severely affects air quality, making it unhealthy for sensitive people. One ton of wet sugarcane contains 150 kg of dry sugar, 125 kg of fibers, and 140 kg of dry tops and leaves not used for production but left in the field to decay or burn [3]. Plant fibers consist of cellulose, hemicellulose, lignin, pectin, wax, and other water-soluble substances [4]. Cellulose can be used to produce paper and packaging, whereas lignin can be utilized to load and release pesticides, pharmaceuticals, and biological macromolecules [5]. Lignin has found several applications, for example, as a bio-adsorbent for wastewater treatments [6], as a lignin-based slow-release fertilizer [7], and in renewable energy, chemicals, and material composites [8]. Additionally, using lignocellulosic biomass as feedstock improves biomass utilization and increases the economic opportunity of biorefinery [9]. However, sugarcane straw is one of the lignocellulosic materials which contain cellulose, hemicellulose, lignin, etc. The fiber content could be obtained via chemical or steam explosion from sugarcane straw to upcycle agriculture waste into biobased molded trays.

Agricultural wastes from non-woody herbaceous plants, including corn stalks, sugarcane bagasse, wheat and rice straw, and cotton stalks, are being valorized to obtain paper pulp for the production of biobased disposable products, thereby promoting waste reduction and the use of renewable resources [10]. Good pulp for papermaking requires fibers that are medium to long, flexible, and high felting power [11]. The pulping process is commonly used to extract cellulose from agricultural waste, such as sugarcane straw, rice straw, wheat straw, and corn stalks. This process involves breaking down the fibrous material of the waste material to extract the cellulose fibers [10]. Several methods of fiber extraction are commonly used, including mechanical pulping, semi-mechanical pulping, chemical pulping, thermomechanical pulping, chemithermomechanical pulping, recycling, and organosolv pulping [12]. Each method has unique advantages and disadvantages, depending on the waste material used and the desired end product. Mechanical pulping involves physically grinding the agricultural waste into fibers, while semi-mechanical pulping combines mechanical and chemical processes to break down the material. Chemical pulping uses sodium hydroxide and sulfur dioxide to dissolve the non-cellulose components and extract the cellulose fibers. Thermomechanical, chemithermomechanical, and organosolv pulping employs heat, chemicals, and organic solvents to extract and break down the fibers, respectively [13].

Steam explosion pulping is a physicochemical treatment that involves using high-pressure steam for short periods, followed by rapidly reducing the pressure, typically used to break down lignocellulosic biomass into its constituent parts, such as cellulose, hemicellulose, and lignin [14]. During the steam explosion process, the biomass material is first exposed to high-pressure steam, which softens and swells the fibers, allowing easier separation of the cellulose, hemicellulose, and lignin [15]. Steam explosion is an attractive method for biomass processing due to its low energy consumption, relatively low cost, and ability to produce high-quality lignocellulosic materials [16]. There are a few studies on the extraction and characterization of sugarcane straw fiber for use as a pozzolanic material [17], a filler/reinforcement of commercial biopolymers [18], and textile fiber [19]. However, a study has yet to be conducted to analyze the improved physical and mechanical properties of sugarcane fiber molded trays using the steam explosion method. This research introduces the novel use of steam explosion pulping to extract lignin and cellulose from sugarcane straw, enabling the production of molded pulp trays with improved physical, mechanical, and water resistance properties. It offers a sustainable solution for upcycling agricultural waste into biobased materials for food containment.

## 2. Materials and Methods

### 2.1. Chemicals

Acetic acid (CH_3_COOH), ethanol (CH_3_OH), sodium chlorite (NaClO_2_), sodium hydroxide (NaOH), and sulfuric acid (H_2_SO_4_) were all procured from RCI Labscan, Bangkok, Thailand. Anthraquinone (AQ) was purchased from Sigma-Aldrich, Saint Louis, MO, USA, and benzene from QREC, Auckland, New Zealand. All the chemicals used in this study were of analytical grade.

### 2.2. Preparation of Sugarcane Straw

Sun-dried straw from sugarcane was acquired from Chai Nat Province, near Bangkok, Thailand. Subsequently, the samples were taken to the Postharvest Technology and Packaging laboratory at Mae Fah Luang University in Chiang Rai, Thailand. The straw−free of dust was then cut into small pieces and subjected to 24 h of drying at a temperature of 70 °C. Finally, the dried sugarcane straw was stored in a polypropylene (PP) bag with a zip-lock at room temperature (25 °C).

### 2.3. Extraction of Sugarcane Straw Fiber Using Chemical and Steam Explosion Pulping Methods

Forty grams of dry sugarcane straw was placed in glass bottles, and then 400 mL of 5%, 10%, and 15% NaOH with 0.2% AQ was added. The pulp was heated at 121 °C for 1 h. The straw was removed from the bottles and rinsed with distilled water until the pH was 7. The yield of fiber was calculated (Equation (3)). Forty grams of dry sugarcane straw was weighed per batch and placed in a steam explosion machine at a pressure of 1.77, 1.96, and 2.16 MPa at 220 °C for 4 min. The STE-processed sugarcane straw samples were washed with distilled water and screened through stainless steel sieve. According to Wang et al. [20], steam explosion has two key factors: temperature and retention time. They introduced a simple factor for evaluating and optimizing the steam explosion process, which defines the severity of steam explosion in terms of the combined effect of temperature and retention time in Equations (1) and (2).

(1) $$R_{0}={te}^{\frac{T-100}{14.75}}$$

where R_0_ is the reaction ordinate, t is the residence time (min), T is the reaction temperature (°C), 100 is the base temperature (°C), and 14.75 is the conventional energy of activation based on a first-order reaction. The index of severity factor (SF) was calculated as follows:

SF = log_10_ R_0_ (2)

The percent yield was determined to estimate the output of fiber from input of straw and analyze the feasibility of the process. The yield (%) was calculated using Equation (3):

(3) $$\%\;\mathrm{yield}=(\frac{W2}{W1})\times 100$$

where W_1_ is the weight of the sample before the pulping process (g), and W_2_ is the weight of the sample after the pulping process (g).

### 2.4. Physicochemical Properties of Sugarcane Straw Fiber (SSF)

#### 2.4.1. Soxhlet Extraction of Extractives from SSF

Extractives were analyzed according to the TAPPI T 204 cm-97 standard. Ten grams of SSF was placed in a filter paper inside a glass tube. Soxhlet equipment was used with 200 mL of ethanol: benzene (ratio 1:2) in a round-bottom flask and heated for 4 h. Afterward, the solvent was separated from the solid using a vacuum filtration machine, model WJ-20 (SIBATA, Saitama, Japan), and ethanol was used to remove the remaining benzene from the solid. The solid was further treated in the Soxhlet apparatus using 200 mL of 95% ethanol for 4 h. It was then filtered with a vacuum filter and rinsed with distilled water. The sample was placed in a 1000 mL Erlenmeyer flask with 500 mL of distilled water and boiled for 1 h. It was then filtered using a vacuum filter and rinsed with 500 mL of hot water. The sample was allowed to cool to room temperature before being dried at 105 °C for 24 h. The extractives were calculated using Equation (4).

(4) $$\mathrm{Extractive}\;(\%)=(\frac{W1-W2}{W1})\times 100$$

where W_1_ is the weight of SSF before extraction (g), and W_2_ is the weight of SSF after extraction (g).

#### 2.4.2. Holocellulose Content of SSF

Holocellulose (the total polysaccharide fraction) was analyzed according to the Method of Wood Chemistry (TAPPI section, 10 January 1946). Extractive-free sample (2.5 g) was placed into a 250 mL Erlenmeyer flask with 150 mL of distilled water, 0.2 mL of pure acetic acid, and 2 g of NaClO_2_. The flask was heated in a water bath at 70 °C, and every hour, 0.2 mL of pure acetic acid and 1 g of NaClO_2_ were added for 4 h. The sample solution was then cooled to below 10 °C for 30 min and filtered through a filtering crucible, washing it with iced distilled water, followed by washing with acetone (approximately 100 mL). The sample was dried at 105 °C for 24 h. The holocellulose content was calculated using Equation (5).

(5) $$\mathrm{Holocellulose}\;(\%)=(\frac{Holocellulose\;(g)}{Extractive\;free\;wood\left(g\right)+Extractive(g\;)})\times 100$$

#### 2.4.3. Lignin Content of SSF

The lignin content was analyzed using the TAPPI T 222 om-02 method. One gram of the extractive-free sample was placed in a 100 mL beaker, and 15 mL of cool sulfuric acid was added. The beaker was covered with a watch glass and placed in an iced bucket, with the temperature controlled at around 20 °C, and stirred every 15 min for 2 h. Then, 400 mL of distilled water was added to the sample in a 1000 mL round-bottom flask, the beaker was rinsed with 3% H_2_SO_4_, and the volume was adjusted to 575 mL with distilled water. The sample was then boiled for 4 h while maintaining the volume of water content. The sample was left overnight before filtering through a filtering crucible, rinsed with hot water, and then dried at 105 °C for 6 h. The lignin content was calculated using Equation (6).

(6) $$\mathrm{Lignin}\;\mathrm{content}\;(\%)=(\frac{Lignin(g)}{Extractive\;free\;wood\left(g\right)+Extractive\;(g)})\times 100$$

### 2.5. Formation of Molded Pulp Trays from the SSF

Molded pulp trays from SSF were formed using a hydraulic hot-press machine model LP-S-80 (Labtech, Samutprakarn, Thailand). In the first step, the fiber was weighed and soaked in water to soften it before blending. Next, the blended fibers were carefully placed in a mold, spread evenly, and then subjected to hydraulic pressing at 1.72 MPa for 3 min to drain excess water. Afterward, the molded fibers were put into the hydraulic hot-press at 120 °C for 7 min at 1.72 MPa.

### 2.6. Physical, Mechanical, and Microstructural Properties of SSF Trays

#### 2.6.1. Grammage of Molded Pulp Trays from the SSF

Samples were prepared according to TAPPI T 400 sp-02, and the grammage was determined following TAPPI T 410 om-08. Paper sheets were cut into 10 × 10 cm^2^ pieces before weighing. The grammage of the molded pulp tray was calculated using Equation (7).

(7) $$\mathrm{Grammage}\;(g/m^{2})=\frac{Weight\;of\;the\;sample\;(g)}{Length\;of\;the\;sample\left(m\right)\times Width\;of\;the\;sample\;(m)}$$

#### 2.6.2. Water Wetting Time and Thickness Swelling of Molded Pulp Trays from the SSF

The water wetting time was measured by dispensing one drop of water from a burette onto a molded pulp tray placed at a height of 1 cm. The time taken for the tray to absorb the water was recorded using the method outlined by Razak et al. [21]. Thickness swelling was measured in accordance with ASTM D 1037-99. The increase in thickness of the molded pulp tray was measured after it was submerged in a water bath at 25 °C for 24 h. Excess water was removed from the tray using tissue paper, and the thickness was measured again. The thickness swelling value was calculated using Equation (8).

(8) $$\mathrm{Thickness}\;\mathrm{swelling}\;(\%)=(\frac{Final\;thickness-Initial\;thickness}{Initial\;thickness})\times 100$$

#### 2.6.3. Color (L* a* b*) Values of Molded Pulp Trays from the SSF

Brightness is a crucial factor for packaging papers and cardboard. To measure the color of the SSF tray, a spectrophotometer model CM-600d (KONICA MINOLTA, Tokyo, Japan) was used, and the data were analyzed using color data software CM-s100w Sectra MagicTM NX lite. The values for L*, a*, and b* were determined.

#### 2.6.4. Crystallinity Index of Molded Pulp Tray

The crystallinity of the SSF trays made from either CP or STE pulp was analyzed using an X-ray diffractometer (XRD) model X’Pert PRO (Philips, Amsterdam, The Netherlands). Samples were scanned over the 2θ range of 5–50° with a scanning speed of 2° min^−1^ (2θ) and a step of 0.02° (2θ) to examine the structures. The crystallinity index was calculated using Equation (9) [22].

(9) $$\mathrm{Crystallinity}\;\mathrm{index}=\frac{I200-Iam}{I200}$$

where I_200_ is the maximum intensity due to reflections of the planes (200) around 23°, and I_am_ is the amorphous contribution, at the minimum intensity between 15° < 2θ < 23°

#### 2.6.5. Tensile and Compression Tests of Molded Pulp Trays from the SSF

Testing was performed to determine the flexibility, strength, and maximum tensile strength. The test slowly increased the tensile strength and recorded the changes that occurred between stress and strain until the specimen was broken. Tensile strength was analyzed according to TAPPI T 494 om-01, and the sample was prepared following TAPPI T 400 sp-02. The sample was placed in a universal testing machine (UTM) model 5566 (INSTRON, Norwood, MA, USA), and then the sample was pulled at a rate of jaw separation of 25 mm/min until failure of the sample. Tensile strength was calculated using Equation (10), and the tensile index was calculated using Equation (11).

(10) $$\mathrm{Tensile}\;\mathrm{strength}\;(\mathrm{kN}/m)=\frac{Max\;load\;(kN)}{Width\;of\;sample\;(m)}$$

(11) $$\mathrm{Tensile}\;\mathrm{index}\;(\mathrm{Nm}/g)=\frac{Tensile\;strength\;(N/m)}{Grammage\;(g/m^{2})}$$

The molded pulp tray was subjected to a compression test using the universal testing machine (UTM) model 5566 (INSTRON, USA) according to TAPPI T 804 om-06. The speed of the machine was set to 13 ± 2.5 mm/min until failure of the sample, and the compression strength and compression index were calculated using Equations (10) and (11), respectively.

(12) $$\mathrm{Compression}\;\mathrm{strength}\;(N/m^{2})=\frac{Max\;load\;(N)}{Length\;of\;sample\left(m\right)\times Width\;of\;sample\;(m)}$$

(13) $$\mathrm{Compression}\;\mathrm{index}\;(N/g)=\frac{compression\;strength\;(N/m)}{Grammage\;(g/m^{2})}$$

#### 2.6.6. Attenuated Total Reflectance Fourier-Transform Infrared (ATR–FTIR) Spectroscopy of SSF and Molded Pulp Tray

Evaluation of the chemical constituents of lignocellulose and confirmation of their chemical structure were performed using ATR–FTIR [20]. ATR-FTIR measurements were performed using a spectrometer model Nicolet iS50 (Thermo Scientific, Waltham, MA, USA) with ATR from a total scan of 32 scans with a resolution of 4 cm^−1^ over the 4000–400 cm^−1^ range.

#### 2.6.7. Scanning Electron and Two-Photon Microscopic Analysis of the Molded Pulp Tray

The morphology of the molded pulp trays was examined using a scanning electron microscope (SEM) model JEOL JSM–6700F (JEOL Ltd., Tokyo, Japan) to observe the arrangement of fibers. Two-photon microscopy was used to obtain a 3D representation of the internal structure of molded pulp trays made from sugarcane straw, following the method outlined by Shiekh et al. [23].

### 2.7. Statistical Analysis

The data were statistically analyzed. Analysis of variance (ANOVA) and Duncan’s test were performed using IBM SPSS Statistics 20. Samples were analyzed at a 95% confidence level (*p* < 0.05) for each treatment. Three replications were analyzed for physicochemical and physical composition and ten replications for mechanical properties.

## 3. Results and Discussion

### 3.1. Effect of Chemical and Steam Explosion Pulping Methods on the Characteristic Properties of Sugarcane Straw Fiber

#### Percent Yield and Component Composition of Sugarcane Straw Fiber

Table 1 presents the percentage yield, extractives, holocellulose, and lignin contents of the sugarcane straw fiber (SSF) samples from different treatments of pulping methods. The methods included chemical pulping (CP) with 5%, 10%, and 15% sodium hydroxide (NaOH) and steam explosion (STE) at different severity factors (SFs) of 3.99, 4.28, and 4.43. The highest yield was obtained from STE at SFs 4.28 and 4.43, with a percentage yield of 59.62% and 54.33% (*p* < 0.05), respectively. The CP with 5% NaOH resulted in a percentage yield of 47.93%, while the CP with 10% NaOH and 15% NaOH resulted in a lower yield of 40.25% and 31.95% (*p* < 0.05), respectively. The highest extractive content was obtained from STE treatments, with values ranging from 22.45% to 25.23%. Among the STE treatments, SF 4.43 resulted in the highest extractive content. In contrast, the extractive content of CP treatments with NaOH ranged from 8.36% to 10.03%, with the lowest amount obtained from CP with 15% NaOH (*p* < 0.05). Additionally, the holocellulose content of the SSF samples varied significantly depending on the treatment method used. Among the CP treatments, the highest amount of holocellulose was obtained in SSF treated with 15% NaOH, with a value of 66.69 ± 1.19% (*p* < 0.05). In contrast, the holocellulose content of STE-treated SSF ranged from 58.01 ± 0.15% to 65.00 ± 0.81% (*p* < 0.05), with the lowest amount obtained in SSF treated with the STE method at an SF of 4.43. Moreover, in CP treatments, the lowest amount of lignin was obtained from CP with 15% NaOH, with a value of 2.93 ± 0.08% (*p* < 0.05). In contrast, the lignin content of STE treatments ranged from 20.65 ± 1.31% to 22.27 ± 2.61% (*p* < 0.05), with the highest amount obtained from STE at SF 3.99.

During the STE pulping, an increment in the severity factor (pressure, temperature, or holding time) tended to decrease SSF yield. However, at lower treatment conditions of STE, the SSF fibers retained their structural integrity compared to those of the CP samples. The least disintegration of fiber obtained from *Cocos nucifera* L. mesocarp employing steam explosion and anaerobic digestion was reported by Inseemeesak and Areeprasert [24]. Sluiter et al. [25] reported that after the STE, some of the ash components in the biomass can be removed during the washing process. The dirt and other inorganic compounds from biomass were removed by the STE process [26,27]. The chemical pulping process encountered a loss of 80% of lignin and 50% of hemicellulose in hydrolyzed Acacia wood chips [28]. According to Boonterm et al. [29], in NaOH pulping of rice straw, a higher concentration of NaOH resulted in lower fiber yield andsmall size of fibers, shorter fiber diameter, and length. At higher concentrations of NaOH, cellulose was degraded which resulted in a significant loss of carbon. Moreover, Jiang et al. [30] reported a reduction in hemicellulose, pectin, and water-soluble matter with an increment in the steam pressure (severity factor). Simangunsong et al. [31] reported that the percent yield loss in pulp increased with the SF.

Inseemeesak and Areeprasert [24] reported that ash and extractive contents were removed by the release of steam during the extraction of fibers from *Cocos nucifera* L. mesocarp. Ewunie et al. [32] reported that the extractive components in pretreated *Jatropha curcas* fruit shell (JCFS) were comparatively lower than the untreated JCFS. The extractives in plant materials consist of lipids, phenolic compounds, terpenoids, fatty acids, resin acids, sterol esters, sterol, and waxes [33]. At relatively lower temperatures (40 °C), the NaOH concentration had more effect on increasing holocellulose content [34]. Han et al. [35] reported that the holocellulose content increased while the lignin content decreased after pressurized steam treatment of wheat straw. Furthermore, lignin content in the CP process depends on NaOH concentration [36]. The yield of tomato stalk fibers was decreased due to lignin removal using NaOH [37]. Hemicellulose and lignin were reported to be more soluble in the matrix at high SFs employed during STE [38]. STE possibly improves the cellulose percentage by eliminating lignin and hemicellulose without using chemical solvents [39,40]. Tanpichai et al. [41] reported that at a higher pressure of STE, lignin content was improved slightly. Meanwhile, longer holding time at high pressure showed repolymerization of lignin [42].

### 3.2. Effects of Treatments on the Physical and Mechanical Properties of SSF Trays

#### 3.2.1. Physical Properties of SSF Trays

**Figure 1:**
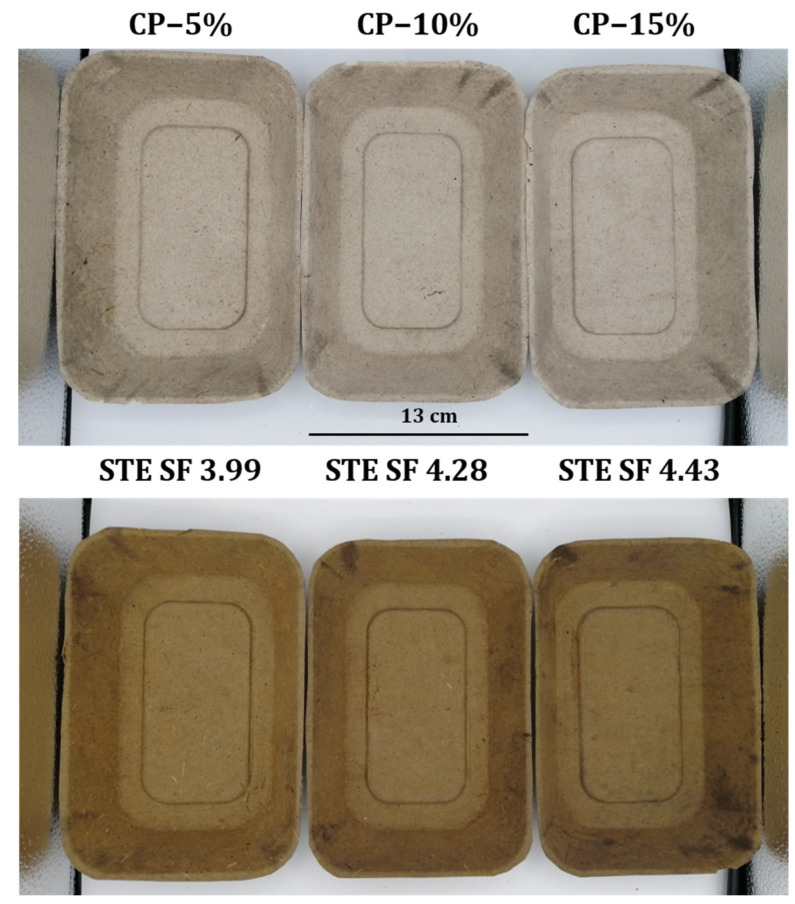
Molded pulp trays from SSF extracted from chemical and steam explosion pulping.

The SSFs from CP and STE were molded using the hydraulic hot-pressing machine to make the trays as shown in Figure 1. The weight, grammage, thickness, water wetting time, thickness swelling, and contact angle results of SSF fabricated trays are provided in Table 2. The weight of the tray samples varied depending on the treatment employed in CP and STE pulping methods. The CP samples treated with 5%, 10%, and 15% NaOH had weights of 17.11 ± 0.21 g, 16.91 ± 0.40 g, and 16.30 ± 0.32 g, respectively. The STE samples treated at SF 3.99, 4.28, and 4.43 had weights of 20.37 ± 1.12 g, 20.30 ± 1.47 g, and 19.80 ± 0.52 g, respectively. The weights of all the CP tray samples did not differ from each other (*p* > 0.05). No difference in the weights of tray samples was observed in the STE pulping method. However, an increment in the weights of STE-processed SSF tray samples was analyzed in comparison to the CP tray samples (*p* < 0.05) due to higher lignin content and other composition remaining in STE fiber as shown in Table 1.

Grammage values of the samples were based on the conditions applied in the CP and STE methods. The CP samples treated with 5%, 10%, and 15% NaOH had grammages of 636.47 ± 13.80 g/m^2^, 611.24 ± 19.55 g/m^2^, and 531.21 ± 20.25 g/m^2^. The STE samples treated at SF 3.99, 4.28, and 4.43 had grammages of 761.36 ± 3.68 g/m^2^, 650.80 ± 30.67 g/m^2^, and 607.09 ± 31.06 g/m^2^. The grammages of the CP samples treated with 5% and 10% NaOH did not differ significantly; however, both samples were higher (*p* < 0.05) in grammage value compared to the 15% NaOH-treated sample in CP. The grammages of the STE at SF 3.99 were higher than the other STE samples (*p* < 0.05).

**Figure 2:**
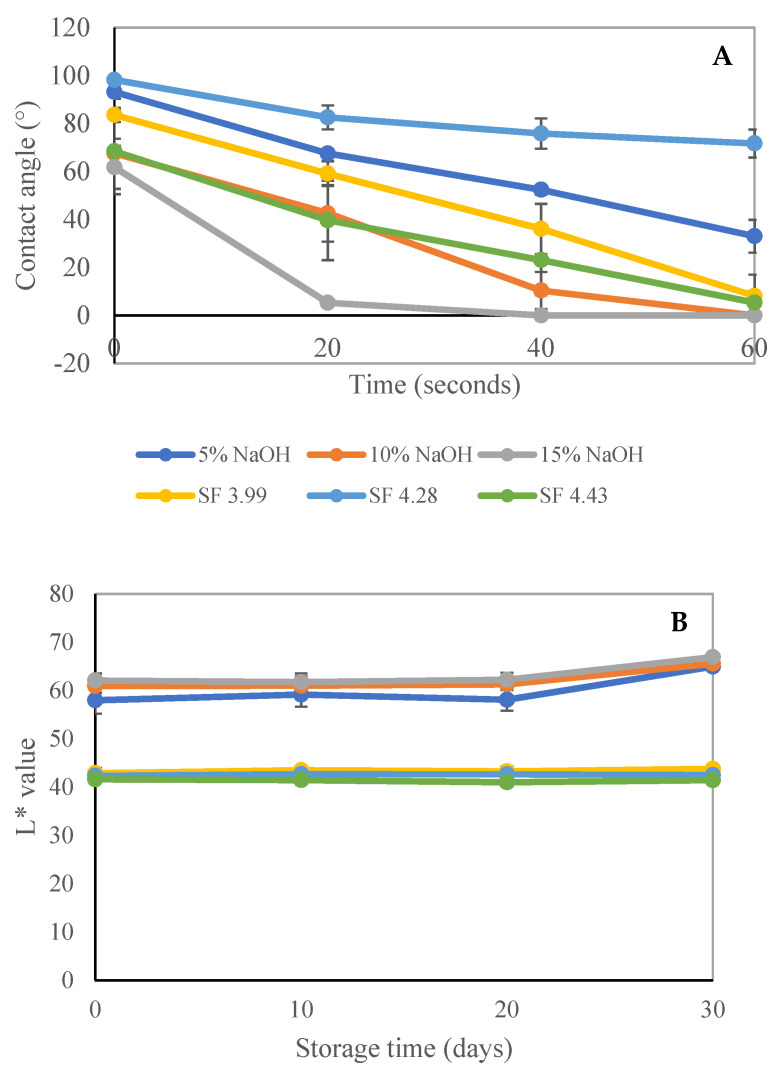

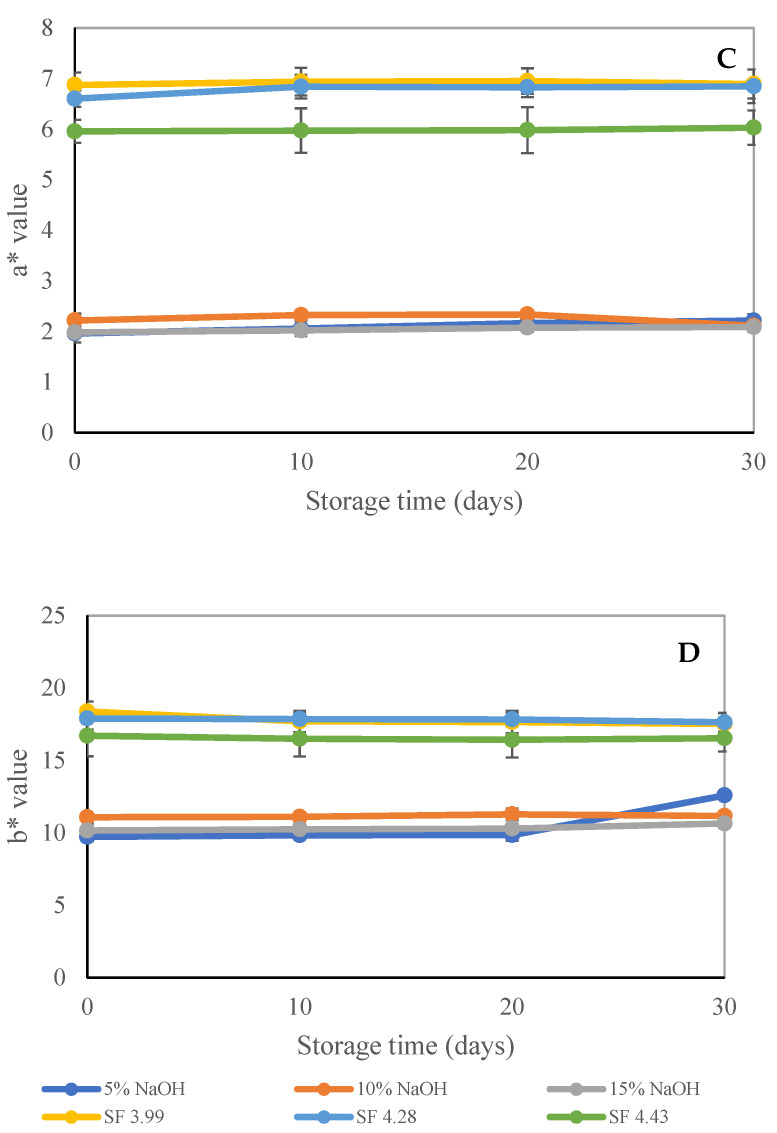
Contact angle (**A**) and color values, L* (**B**), a* (**C**), b* (**D**) of trays fabricated from SSF extracted from chemical and steam explosion pulping at 25 °C of storage. The error bars represent the standard deviation. SSF: sugarcane straw fiber; CP: chemical pulping; STE: steam explosion pulping, SF: steam explosion severity factor.

The lowest water wetting time was observed for the sample treated with 15% NaOH (0.99 ± 0.13 s) (*p* < 0.05), while the highest water wetting time was observed for the sample treated with steam explosion at SF 4.28 (6.72 ± 0.70 s) (*p* < 0.05). These results suggest that the use of 15% NaOH as a sample treatment can significantly reduce the water wetting time of the material caused by removing lignin, while the use of steam explosion at SF 4.28 can significantly increase the water wetting time. These findings have practical implications for industries that deal with materials that need to be water-resistant or water-repellent. Additionally, the lowest thickness swelling was observed for the sample treated with steam explosion at SF 4.28 (3.24 ± 1.13%) (*p* < 0.05), while the highest thickness swelling was observed for the sample treated with 15% NaOH (36.62 ± 4.05%) (*p* < 0.05). The results revealed that the use of steam explosion at SF 4.28 as a sample treatment can significantly reduce the thickness swelling of the SSF tray sample, while the use of 15% NaOH can significantly increase the thickness swelling. The steam explosion process was found to affect the water properties of rice straw by decreasing its hydrophilic nature after the process [35]. The higher water resistance of SSF trays was further analyzed by the contact angle of the SSF tray samples prepared by the CP and STE pulping methods. Figure 2A shows the contact angle measurements of SSF molded pulp trays. The contact angle of SSF trays from STE at SF 4.28 was found to be higher than that of CP trays. In the CP treatment, the highest contact angle was observed for the sample treated with 5% NaOH. This can be attributed to the remaining lignin in the SSF, which leads to high hydrophobic properties in the trays. The highest contact angle of 88° was reported in a lignin-fortified cellulose nanofiber film with significant hydrophobic properties [43]. Therefore, the STE treatment at SF 4.28 showed the best water resistance, which can be attributed to the increased lignin content resulting in higher water wetting time (min) and less thickness swelling in the SSF trays, as shown in Table 2.

While Figure 1 shows the appearances of the trays, the color values (L*, a*, b*) of trays from CP and STE under different treatments of NaOH and SF are represented in Figure 2B–D. The whiteness (L*) of STE-processed SSF trays was lower than that of CP at day 0 of storage (Figure 2B). Additionally, higher red (a*) (Figure 2C) and yellow (b*) (Figure 2D) color values were also obtained in STE in comparison to the CP SSF tray samples during the first day of storage. The results of the comparison of the brightness of CP and STE trays from SSF showed that CP was brighter than STE trays and the color values changed insignificantly until 30 days of storage in all CP and STE treatments. The white color of trays produced through CP is attributed to the bleaching effect of anthraquinone used during SSF isolation. STE trays exhibit lower whiteness due to the mild carbonization and chemical breakdown of cell wall constituents, resulting in a darker brown hue. Anthraquinone in CP and the carbonization process in STE contribute to the color differences observed in the trays. Consequently, the SSF carbon content increased in trays processed with STE, giving it the previously reported dark brown hue as described by Inseemeesak and Areeprasert [24]. The white color of the alpha−cellulose was affected by the amount of non-cellulosic components like lignin and hemicellulose [44]. The color of the exploding fiber materials was a result of the chemical breakdown of cell wall constituents, particularly lignin and wood extractives, at extremely high temperatures. Furfural and hydroxymethyl furfural can react with hydrolyzed hemicellulose and depolymerized lignocellulosic components such as tannins, flavonoids, or solubilized sugars, to form large molecules that have a brown color [41].

#### 3.2.2. Mechanical Properties of Fabricated Trays

The tensile strength, tensile index, elongation, compression strength, and compression index of samples treated with different concentrations of NaOH and the STE process at different severity factors (SFs) are presented in Table 3. The tensile strength of the CP–5% NaOH treatment showed the highest value of 4.03 ± 0.33 kN/m, which was significantly different from the CP–10% NaOH (1.89 ± 0.60 kN/m) and CP–15% NaOH (1.27 ± 0.06 kN/m) treatments. On the other hand, the STE at SF 4.28 treatment showed the highest tensile strength of 7.32 ± 1.69 kN/m, which also differed significantly from the other two STE treatments (STE at SF 3.99: 4.60 ± 0.61 kN/m and STE at SF 4.43: 4.03 ± 0.43 kN/m). In terms of the tensile index, which is an indicator of material strength in an SSF tray, the highest value was obtained from the STE at SF 4.28 treatment (11.24 ± 2.60 Nm/g), which was significantly different from all other treatments. The CP-5% NaOH treatment showed the second-highest tensile index (6.33 ± 0.53 Nm/g) (*p* < 0.05), compared to the other two NaOH treatments (CP–10% NaOH: 3.08 ± 0.99 Nm/g and CP–15% NaOH: 2.38 ± 0.11 Nm/g). In addition, elongation values of the CP–5% NaOH treatment showed the highest value of 0.75 ± 0.06 mm (*p* < 0.05), compared to the CP–10% NaOH (0.45 ± 0.03 mm) and CP–15% NaOH (0.40 ± 0.02 mm) treatments. The STE at SF 4.28 treatment showed the highest elongation value of 0.49 ± 0.03 mm, which was significantly different from the other two STE treatments (STE at SF 3.99: 0.43 ± 0.08 mm and STE at SF 4.43: 0.29 ± 0.04 mm). Overall, the results suggest that the STE treatment at SF 4.28 was the most effective in improving the material strength of the SSF tray, as it showed the highest tensile strength and tensile index values, as well as a relatively high elongation value. The CP–5% NaOH treatment also showed relatively good results in terms of tensile strength and the tensile index, but its elongation value was higher than the STE treatments. The CP–10% and CP–15% NaOH treatments showed lower values for all three parameters compared to the other treatments, indicating their lower effectiveness in improving mechanical strength. Overall, the hypothesis about trays produced under SF 4.28 showed improved mechanical strength compared to CP treatments.

Furthermore, the treatments with varying concentrations of sodium hydroxide (CP–5% NaOH, CP–10% NaOH, and CP–15% NaOH) show a clear trend of decreasing compression strength and compression index with an increasing concentration of sodium hydroxide. This trend is observed likely because higher concentrations of NaOH can cause greater degradation of the fibers, leading to weaker materials. In contrast, the steam explosion treatments show a more complex relationship between the severity factor of compression strength and the compression index. The STE at SF 4.28 treatment had the highest compression strength and compression index values, whereas the STE at SF 3.99 treatment had the lowest values. However, the STE at SF 4.43 treatment had intermediate values. It is possible that the steam explosion process can lead to changes in the fiber morphology and structure, which can affect the mechanical properties of the SSF trays. Gu [45] reported that a high concentration of the NaOH solution led to the increased leaching of lignin, pectin, fatty acids, and cellulose from the fiber, which can ultimately weaken the strength of the coir fibers. A high NaOH concentration also increased the delignification process, which may have caused damage to the structure of the *Cyperus odoratus* fiber [46]. Han, Cheng, Deng, Dai, Zhang and Wu [35] reported that straws treated for 10 min with STE showed lower tensile properties than straws treated for 5 min at the same pressure. According to the report, the pressurized steam explosion caused structural changes in the lignin of Kenaf bast [47]. The tensile strength (TS) measurements of unbleached, oxygen-delignified, and fully bleached pulp sheets revealed that the strength of the lignin-free pulp sheets was diminished as a result of localized fiber degradation [48]. Simangunsong, Ziegler-Devin, Chrusciel, Girods, Wistara and Brosse [31] reported that the small size of particles increased with the severity factor. Boonterm, Sunyadeth, Dedpakdee, Athichalinthorn, Patcharaphun, Mungkung and Techapiesancharoenkij [29] reported that NaOH and STE−induced changes in the cellulose fiber in rice straw.

### 3.3. Fourier-Transform Infrared Spectroscopy (ATR-FTIR) and Crystallinity Index (CI) of SSF Trays

**Figure 3:**
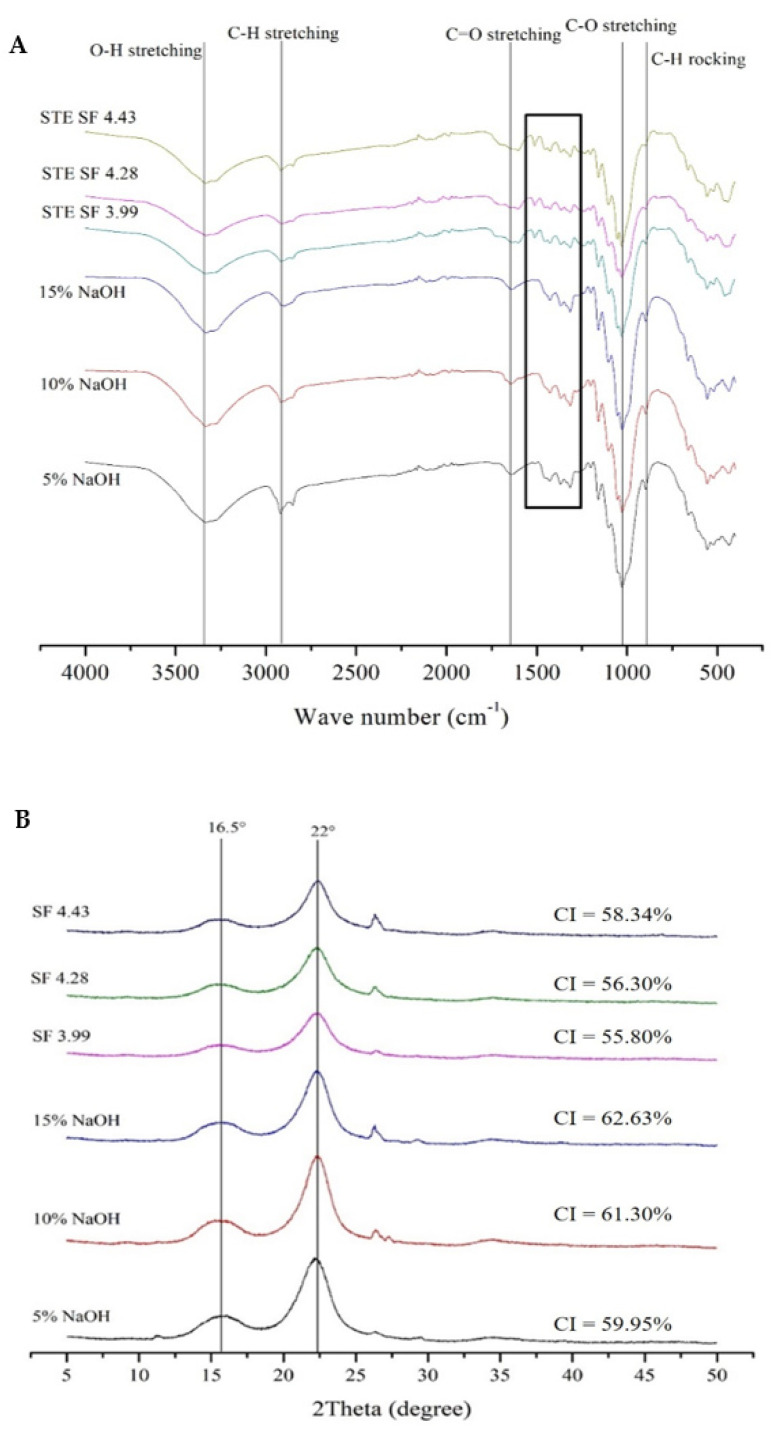
ATR-FTIR spectra (**A**) and diffractograms (**B**) of trays fabricated from SSF extracted from chemical and steam explosion pulping methods. STE: steam explosion pulping, SF: steam explosion severity factor.

The ATR-FTIR and CI results of the CP– and STE–processed trays are presented in Figure 3. Figure 3A displays the ATR-FTIR spectra of SSF trays from CP and STE. The ATR-FTIR spectra can be utilized to evaluate the chemical constituents of lignocellulose and confirm their chemical structure. Holocellulose is composed of hemicellulose and cellulose, which together form an amorphous shell surrounding the cellulose portion. Hemicellulose is softer compared to cellulose. The peak at 3331 cm^−1^ in the spectra corresponds to the hydroxyl group ‘O–H stretching frequency’ and is indicative of the adsorbed water. All fiber samples showed the 3331 and 2920 cm^−1^ absorption bands associated with the hydroxyl and cellulose C–H stretching, which could validate the amount of lignin and hemicellulose present in the SSF trays from STE but not in the CP method. This result was in agreement with the chemical composition measurements (Table 1). Only trays from CP showed the peak at 1640 cm^−1^, which is related to the absorption of water. The peak at 1429 cm^−1^ is associated with the frequency of CH or OH bending in cellulose. The trays from CP and STE displayed the important peaks that represented cellulose. The C–H deformation vibration frequency and the O–H vibration frequency in cellulose are related to the peaks at 1366–1368 and 1316 cm^−1^, respectively. ATR-FTIR can qualitatively show the cellulose crystallinity index. The O–CH in-plane bending vibration at 1430 cm^−1^ and the C–H bending vibration at 1368 cm^−1^ are particularly sensitive to the cellulose crystalline structure; the broadening of these bands reflects a higher proportion of the crystalline structure. Furthermore, the aforementioned results are validated with the previously obtained ATR-FTIR results for SSF tray samples.. The SSF trays from STE also displayed the absorption peaks for lignin at 1603, 1512, 1273, and 1223 cm^−1^ [49], which were absent in the trays from CP. Moreover, the peak at 1238 cm^−1^ attributed to the guaiacyl in lignin [44,50] was also present in the tray from STE. The amorphous area of cellulose was associated with the cellulose C–H rocking at 900 cm^−1^ and the C–H stretching vibrations at 2919 cm^−1^ [50].

The degree of the crystallinity index (CI) was determined from the intensity of the crystalline peak located at 2θ of ~22° and the minimum intensity of the peaks located between 16.5° and 22.6°, corresponding to the amorphous area, as shown in Figure 3B. The results showed that the increase in NaOH concentration and the severity factor in both CP and STE methods led to an increase in CI peaks. The highest CI value was observed at 15% NaOH. The elongation of the SSF tray samples was found to be related to the crystallinity index (CI), which provided strength but also caused brittleness and decreased elongation when CI increased. Based on the tensile strength and elongation at break tests, it can be concluded that the SSF tray from STE is high in strength but low in elasticity, while the tray from CP exhibits moderate strength and elasticity.

Romruen et al. [51] reported that the alkaline extraction of corn cob resulted in the highest crystallinity index (69.45%) due to the reduction of amorphous regions on cellulose fibers, making them closely resemble commercial cellulose. Meléndez-Hernández, Hernández-Beltrán, Hernández-Guzmán, Morales-Rodríguez, Torres-Guzmán and Hernández-Escoto [44] found that increasing the NaOH concentration also increased CI values. Under severe conditions, hemicellulose degradation and amorphous cellulose removal made it easier for alkali to penetrate the cellulose, leading to decreased crystallinity and a reduced crystallite size [50]. The STE process can also affect the CI of SSF, as reported by Tanpichai, Witayakran and Boonmahitthisud [41] who found an increase in CI in pineapple leaf fibers after STE due to the removal of amorphous portions. Similarly, Ref. [52] found an improvement in CI for banana fibers after mechanical treatment through high-pressure homogenization and higher-output-power ultrasonication, which led to the degradation of amorphous cellulose. However, a higher SF was found to decrease CI due to cellulose degradation [52].

### 3.4. Impact of Chemical and Steam Explosion Pulping on the SSF Microstructure of Trays Evidenced by Scanning Electron and Two-Photon Microscopy

**Figure 4:**
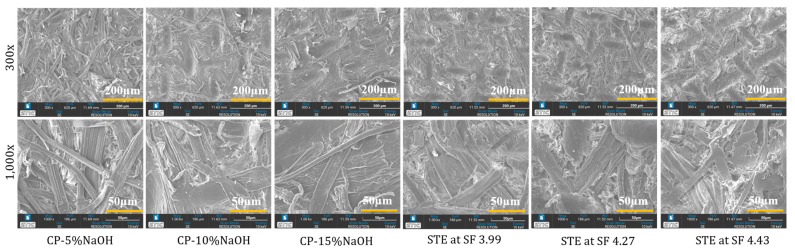
Scanning electron micrographs (300× and 1000×) of trays fabricated from SSF extracted from chemical and steam explosion pulping methods. CP: chemical pulping; STE: steam explosion; SF: severity factor.

Figure 4 displays scanning electron microscopic (SEM) images of the SSF tray at 300× and 1000× magnifications, revealing the morphological characteristics. The results demonstrate that the size of small particles in the tray decreased with increasing NaOH concentration in CP and an increasing SF in STE. When comparing the CP and STE methods, the fibers in the STE trays appeared smaller than those in CP. The SEM images depicted the fibers in the CP trays, while the STE trays showed fibers mixed with other components such as extractives and lignin. Higher NaOH concentration in CP led to extensive chemical extraction, resulting in a shorter fiber diameter and length. The STE method appeared to induce more fiber breakdown, resulting in shorter fiber lengths but larger diameters. In general, microfibers were observed bonded with lignin, and after STE, fibers were extracted from the bundle in smaller sizes. Increasing pressure in STE had a significant impact on reducing both fiber length and diameter.

Sari et al. [53] noted that alkali treatment can remove hemicellulose and decrease fiber diameter. Similarly, Simangunsong, Ziegler-Devin, Chrusciel, Girods, Wistara and Brosse [31] found that increasing SF led to an increase in the size of small particles. When SF was below 3.00, the loss in yield was minimal with small particle sizes, but at a higher SF, there was a significant increase in yield loss with a high concentration of small particles. Boonterm, Sunyadeth, Dedpakdee, Athichalinthorn, Patcharaphun, Mungkung and Techapiesancharoenkij [29] observed that rice straw fibers treated with NaOH and STE differed in their characteristics. The morphology of fibers was altered after STE in the fiber extraction from pineapple leaves, as reported by Tanpichai, Witayakran and Boonmahitthisud [41]. Additionally, Simangunsong et al. [31] reported that a high SF affected the yield loss and particle size in fiber extraction from beech wood.

**Figure 5:**
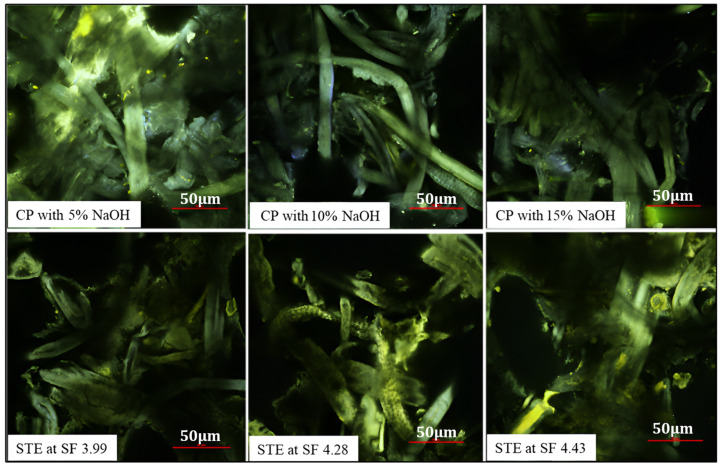
Images from two-photon microscopy; brighter yellow zone represents the fluorescence of lignin. CP shows a clearer shape of fiber because of lignin removal. CP: chemical pulping; STE: steam explosion pulping; SF: steam explosion severity factor.

Figure 5 presents two-photon micrographs of SSF trays prepared using the CP and STE extraction methods. In the CP sample treated with 5% NaOH, a matrix structure containing extractives and lignin was observed. With increased NaOH concentration (10% to 15%), better fiber separation was visible. NaOH treatment shows higher removal of extractives, contributing to improved matrix formation. Conversely, the STE treatment retains more extractives and lignin, resulting in a cellulose fiber matrix with visible fiber aggregation and amorphous regions. The STE samples at SF 4.28 exhibit shorter and thicker fibers with pronounced autofluorescence spots. These results suggest the presence of fluorescent lignin derivatives in certain regions of the fiber matrix. As reported by Chimenez et al. [54], the autofluorescence observed from unbleached samples of sugarcane bagasse further supports this idea, as the emission from fiber walls containing the lignin fraction was longitudinally oriented. The steam explosion pulping method retained lignin and other extractives in the SSF samples, confirming their presence in the SSF fabricated trays.

## 4. Conclusions

Steam explosion (STE) as an ecofriendly technique compared to the chemical pulping process showed promising results for the preparation of sugarcane straw fiber (SSF). The percent yield, content of extractive, and lignin content of SSF prepared by STE were higher than those produced by chemical pulping (CP); however, holocellulose content decreased as the severity factor increased in the STE treatment. The SSF molded pulp trays from STE had high tensile strength and a high-water barrier compared to those from CP but lower compression strength, elasticity, and crystallinity index. The SSF tray from STE was high in strength but low in elasticity, while the tray from CP exhibited moderate strength and elasticity. The micrographs from scanning electron microscopy and two–photon microscopy displayed smaller and shorter dimensions of the STE–derived SSF than that of CP. The color of SSF molded pulp trays from STE was darker than those from CP. The best condition for STE in preparing SSF molded pulp trays was 20 kg/cm^2^ at 220 °C for 4 min or a severity factor (SF) of 4.28, while the best condition for chemical pulping in this study was using 5% NaOH at 121 °C for 1 h. The SSF molded pulp trays from STE could be suitable for producing packages that require high tensile strength and a water barrier, whereas those from CP are suitable for producing packages that require high elasticity and compression resistance, with a whitish color appearance.

## Figures and Tables

**Table 1:** Percent yield and component composition of sugarcane straw fiber.

Sample Treatments	% Yield (db.)	% Extractive	% Holocellulose	% Lignin
Sugarcane straw	–	19.48 ± 0.58	80.52 ± 0.12	22.48 ± 0.14
CP-5% NaOH	47.93 ± 1.19 ^c^	10.03 ± 1.71 ^a^	62.71 ± 0.4 ^b^	6.52 ± 0.75 ^b^
CP-10% NaOH	40.25 ± 1.93 ^b^	8.55 ± 0.79 ^a^	65.99 ± 0.71 ^c^	3.30 ± 0.23 ^a,b^
CP-15% NaOH	31.95 ± 0.96 ^a^	8.36 ± 1.08 ^a^	66.69 ± 1.19 ^c^	2.93 ± 0.08 ^a^
STE at SF 3.99	58.21 ± 0.71 ^e^	24.65 ± 0.73 ^b^	62.46 ± 1.83 ^b^	22.27 ± 2.61 ^c^
STE at SF 4.28	59.62 ± 2.89 ^e^	22.45 ± 2.25 ^b^	65.00 ± 0.81 ^c^	20.65 ± 1.31 ^c^
STE at SF 4.43	54.33 ± 1.12 ^d^	25.23 ± 1.36 ^b^	58.01 ± 0.15 ^a^	21.00 ± 3.37 ^c^

Values are the mean ± the standard deviation (*n* = 5). Different superscripts within the same column followed by different letters (a–e) indicate a significant difference (*p* < 0.05).

**Table 2:** Physical properties of trays using SSF extracted from chemical and steam explosion pulping methods.

SS Treatments	Weight/Tray (g)	Grammage (g/m^2^)	Thickness (mm)	Water Wetting Time (min)	Thickness Swelling (mm)
CP-5% NaOH	17.11 ± 0.21 ^a^	636.47 ± 13.80 ^b,c^	0.73 ± 0.04 ^a^	4.94 ± 0.11 ^d^	16.57 ± 3.48 ^b^
CP-10% NaOH	16.91 ± 0.40 ^a^	611.24 ± 19.55 ^b,c^	0.78 ± 0.08 ^a,b^	3.31 ± 0.20 ^c^	22.94 ± 2.46 ^c^
CP-15% NaOH	16.30 ± 0.32 ^a^	531.21 ± 20.25 ^a^	0.75 ± 0.07 ^a,b^	0.99 ± 0.13 ^a^	36.62 ± 4.05 ^d^
STE at SF 3.99	20.37 ± 1.12 ^b^	761.36 ± 3.68 ^d^	0.93 ± 0.04 ^d^	2.83 ± 0.34 ^bc^	12.12 ± 2.29 ^b^
STE at SF 4.28	20.30 ± 1.47 ^b^	650.80 ± 30.67 ^c^	0.86 ± 0.04 ^c^	6.72 ± 0.70 ^e^	3.24 ± 1.13 ^a^
STE at SF 4.43	19.80 ± 0.52 ^b^	607.09 ± 31.06 ^b^	0.83 ± 0.10 ^b,c^	2.30 ± 0.16 ^b^	21.85 ± 1.48 ^c^

Values are the mean ± the standard deviation (*n* = 5). Different superscripts within the same column followed by different letters (a–e) indicate a significant difference (*p* < 0.05). SS: sugarcane straw; CP: chemical pulping; STE: steam explosion pulping; SF: steam explosion severity factor.

**Table 3:** Mechanical properties of trays using SSF extracted from chemical and steam explosion pulping methods.

Sample Treatments	Tensile Strength (kN/m)	Tensile Index (Nm/g)	Elongation (mm)	Compression Strength (N/m^2^)	Compression Index (N/g)
CP-5% NaOH	4.03 ± 0.33 ^b^	6.33 ± 0.53 ^b^	0.75 ± 0.06 ^d^	17579.1 ± 491.13 ^b^	27.62 ± 0.77 ^d^
CP-10% NaOH	1.89 ± 0.60 ^a^	3.08 ± 0.99 ^a^	0.45 ± 0.03 ^b^	12,887.66 ± 1062.58 ^c^	21.08 ± 1.74 ^c^
CP-15% NaOH	1.27 ± 0.06 ^a^	2.38 ± 0.11 ^a^	0.40 ± 0.02 b ^b,c^	10,055.92 ± 998.6 ^a^	18.93 ± 1.88 ^c^
STE at SF 3.99	4.60 ± 0.61 ^b^	5.84 ± 0.77 ^b^	0.43 ± 0.08 ^b,c^	9855.04 ± 500.26 ^a^	12.51 ± 0.63 ^a^
STE at SF 4.28	7.32 ± 1.69 ^c^	11.24 ± 2.60 ^c^	0.49 ± 0.03 ^c^	10,449.6 ± 1685.32 ^a^	16.06 ± 2.59 ^b^
STE at SF 4.43	4.03 ± 0.43 ^b^	6.94 ± 0.74 ^b^	0.29 ± 0.04 ^a^	9150.85 ± 608.17 ^a^	15.77 ± 1.05 ^b^

Values are the mean ± the standard deviation (*n* = 5). Different superscripts within the same column followed by different letters (a–d) indicate a significant difference (*p* < 0.05).

## Data Availability

All the data are available within this manuscript.
